# Quantitative Determination of Unbound Piperacillin and Imipenem in Biological Material from Critically Ill Using Thin-Film Microextraction-Liquid Chromatography-Mass Spectrometry

**DOI:** 10.3390/molecules27030926

**Published:** 2022-01-29

**Authors:** Robert Włodarski, Karolina Żuchowska, Wojciech Filipiak

**Affiliations:** 1 Department of Anaesthesiology and Intensive Care, 10th Military Research Hospital and Polyclinic, 85-681 Bydgoszcz, Poland; robert.wlodarski@10wsk.mil.pl; 2 Department of Pharmacodynamics and Molecular Pharmacology, Faculty of Pharmacy, Collegium Medicum in Bydgoszcz, Nicolaus Copernicus University in Torun, 87-100 Torun, Poland; karolina.zuchowska@doktorant.umk.pl

**Keywords:** β-Lactam antibiotics, imipenem, piperacillin, ventilation-associated pneumonia (VAP), therapeutic drug monitoring (TDM), personalized therapy, solid-phase microextraction (SPME), thin-film microextraction (TFME), liquid chromatography-mass spectrometry (LC-MS), method validation

## Abstract

β-Lactam antibiotics are most commonly used in the critically ill, but their effective dosing is challenging and may result in sub-therapeutic concentrations that can lead to therapy failure and even promote antimicrobial resistance. In this study, we present the analytical tool enabling specific and sensitive determination of the sole biologically active fraction of piperacillin and imipenem in biological material from the critically ill. Thin-film microextraction sampling technique, followed by rapid liquid chromatography–tandem mass spectrometry (LC-MS/MS) analysis, was optimized and validated for the quantitative determination of antibiotics in blood and bronchoalveolar lavage (BAL) specimens collected from intensive care unit (ICU) patients suffering from ventilation-associated pneumonia (n = 18 and n = 9, respectively). The method was optimized and proved to meet the criteria of US Food and Drug Administration (FDA) guidelines for bioanalytical method validation. Highly selective, sensitive, accurate and precise analysis by means of thin-film microextraction–LC-MS/MS, which is not affected by matrix-related factors, was successfully applied in clinical settings, revealing poor penetration of piperacillin and imipenem from blood into BAL fluid (reflecting the site of bacterial infection), nonlinearity in antibiotic binding to plasma-proteins and drug-specific dependence on creatinine clearance. This work demonstrates that only a small fraction of biologically active antibiotics reach the site of infection, providing clinicians with a high-throughput analytical tool for future studies on personalized therapeutic drug monitoring when tailoring the dosing strategy to an individual patient.

## 1. Introduction

Severe bacterial infections, particularly those caused by multi-drug-resistant (MDR) strains, are the leading causes of morbidity and mortality in patients admitted to intensive care units (ICU). Amongst them, ventilation-associated pneumonia (VAP) occurs in 9–27% of mechanically ventilated (MV) patients at ICUs and its mortality ranges from 30% to 70% [1]. A noticeably wide range of VAP mortality rates results from numerous factors, inter alia various types of ICU wards, along with the different diagnostic criteria and treatment regimes used there, late-onset infection and the pathogen itself, whereby *Acinetobacter baumanii*, *Pseudomonas aeruginosa*, *Enterobacteriaceae* (*Klebsiella pneumoniae, Escherichia coli*) and methicillin-resistant *Staphylococcus aureus* (MRSA) are the most prevalent [2,3,4]. Antibiotic-resistant species are a constantly growing threat in nosocomial infections, with *Klebsiella pneumoniae* being the most prominent example. To de-escalate the problem of resistance, the use of an antimicrobial class and dose adequate for the causative pathogen is recommended [5,6,7]. Both piperacillin [8,9] and imipenem [10], with their broad spectrum of action (Gram-positive, Gram-negative and anaerobic bacteria), are a common choice for the empirical and targeted treatment of critically ill patients. However, the effective dosing of β-lactam antibiotics in these patients is challenging due to the substantial pharmacokinetic (PK) variability, resulting in sub-therapeutic concentrations that may lead to therapy failure and even promote resistance. The altered PK of β-lactams may be related to the increased renal clearance or renal replacement therapy, but it may also result from the disease pathophysiology in critically ill patients, as the high intra- and inter-patient variability of piperacillin concentrations were also reported for individuals without severe renal dysfunctions [11].

To maintain the near-maximal antimicrobial effect of β-lactams, therapeutic drug monitoring (TDM) has been proposed in recent years to individualize dosing strategies. However, to ensure optimal treatment, it is essential to consider additional factors. Firstly, the penetrance of drugs into the pulmonary epithelial lining fluid (ELF), reflecting the site of infection in VAP patients, which is influenced by, among others, pH, lipophilicity, alveolar-capillary membrane, and bronchial inflammation. Consequently, even drugs of the same class, for instance, cefepime, piperacillin or ceftaroline (all being β-lactams), may have considerably different levels of penetrance in the ELF [12]. Secondly, given that only the unbound fraction of antibiotics is responsible for bacteria eradication, determination of the unbound rather than total drug concentration is valid. Importantly, substantial differences between unbound concentrations that are measured vs. recalculated from total concentrations using published protein-binding values were demonstrated [13]. Finally, the economic aspect needs to be considered. Despite the continuously increasing evidence that TDM is a useful strategy to optimize drug exposure [14] and improve clinical outcome [15,16], particularly when pathogens with reduced sensitivity to antibiotics are present [5,17], the poor availability of analytical techniques enabling the TDM of β-lactams, as well as the time until results are available, were reported as frequent obstacles in regular practice [18].

Given the abovementioned reasons, an effective method is being sought for the rapid determination of the dynamically changing concentration profiles of antibiotics in biological material. The applications reported to date are based on high-performance liquid chromatography (HPLC) coupled with ultraviolet (UV) [19,20,21] or mass-spectrometry (MS) detection [22,23,24,25,26]. However, most of them require time-consuming sample preparation and a relatively long run time, which limits their usefulness in daily clinical practice. Moreover, with rare exceptions [13], most studies focused on the determination of total antibiotic concentration, instead of the unbound fraction.

Therefore, the presented work aimed to develop an easy-to-handle, fast analytical method for the quantitative determination of unbound β-lactams (piperacillin and imipenem) in biological samples, enabling the estimation of a degree of antibiotic penetration into the site of infection in ICU patients suffering from ventilation-associated pneumonia. For this purpose, thin-film solid phase microextraction (TFME) was chosen as a sample preparation technique, followed by rapid LC-MS/MS analysis. TFME is based on an equilibrium partition of the free analyte between the sample matrix and solid phase [27], allowing for the high-throughput preparation of 96 samples at once, while the selection of appropriate adsorbents ensures a highly reproducible analysis of even trace levels of diverse analytes in a complex matrix [28]. All TFME blades used in this study were self-made, and the extraction conditions (such as adsorbent type, extraction time, desorption solution, desorption time), as well as the LC-MS/MS conditions (including column type, gradient of mobile phases, MS ionization), were optimized for piperacillin and imipenem. This newly established method was validated according to the US Food and Drug Administration (FDA) guidelines for bioanalytical method validation from 2018 [29] and applied to the analysis of piperacillin and imipenem in biological samples gathered from VAP patients.

## 2. Results and Discussion

Although TDM is an indispensable tool to control the effectiveness of therapy, especially among the critically ill, it is still not implemented globally [30]. Over the last decade, researchers have attempted to create analytical methods for TDM [19,20,21,22,23,31,32], but none of them allowed for the preparation of a large number of samples at the same time. For example, in the method developed by Wolff et al. [21], the sample preparation technique is protein precipitation, in which each sample should be prepared individually according to the developed procedure. Using TFME, it is possible to prepare as many as 96 samples simultaneously in one 96-well plate, which can be immediately placed in an autosampler for rapid LC-MS/MS analysis [33]. Due to the wide range of available sorbents and the development of sampling equipment, TFME has potential applications in many medical specialties, especially in diagnostics [34]. Importantly, only an unbound fraction of antibiotics present in a sample is extracted with the technique applied here, precluding a bias to appear related to the estimation of unbound concentrations from the literature data. In this study, we describe a method that allows for the simultaneous, fast, quantitative determination of piperacillin and imipenem.

### 2.1. LC-MS Conditions Optimization

#### 2.1.1. Chromatographic Column Selection

When the Acquity UPLC^®^ BEH C18 1.7 µm and the Kinetex^®^ 1.7 µm F5 columns were used, the chromatographic separation was incomplete and target analyte peaks were overlapped, regardless of the applied gradient of mobile phases. The best separation of analytes was obtained using column Kinetex^®^ 2.6 µm C18 100 Å 100 mm × 3 mm.

#### 2.1.2. Mobile Phase Gradient Selection

Five different gradients of mobile phases were tested: three of them resulted in incomplete separation and reduced peak areas of analytes. Since the detector response for pure standards was much lower for imipenem compared to piperacillin (difference of two orders of magnitude for the same concentration), efforts were focused on the optimization of imipenem analysis. In this respect, the most satisfactory chromatographic separation was obtained using the gradient elution described in Table 1.

#### 2.1.3. MS Conditions Optimization

For the optimal electrospray ionization in positive mode, the following values were selected for ion source parameters: nebulizing gas flow of 3 L/min, heating gas flow of 10 L/min, drying gas flow 5 mL/min, interface temperature of 300 °C, desolvatation line temperature of 150 °C and heat block temperature of 400 °C. To ensure the accurate identification and quantitation of analytes, the multiple reaction monitoring (MRM) mode was used for MS data acquisition. The appropriate parent ion and product ions for each analyte were chosen and the collision energies were first selected in autotune, followed by manual adjustment. The optimal MRM parameters and transitions for each compound are listed in Table 2.

#### 2.1.4. Quality Control of TFME Blades Preparation

**Figure 1:**
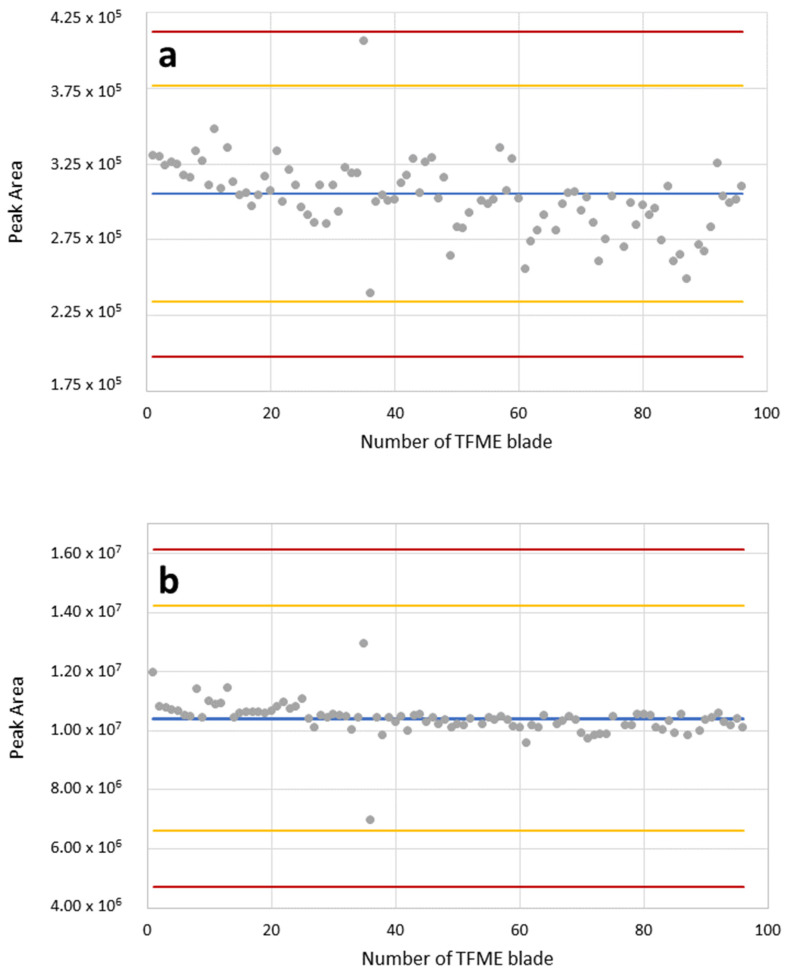
Shewhart charts for QC of self-made TFME blades coated with DVB for imipenem (**a**) and piperacillin (**b**) extraction at concentration 0.1 mg/L. Determined peak areas for all blades are given as grey points. Red lines denote control limit (± 3σ), yellow lines denote warning limits (± 2σ) and the blue line indicates mean peak area for all blades.

Shewhart control charts showed that the extraction of imipenem and piperacillin (Figure 1) from all 96 DVB blades (i.e., complete brush dedicated to 96-well plate) yields reproducible results within the range defined by the warning limits (+/−3σ). Noticeably, the areas integrated under the chromatographic peaks of imipenem are substantially smaller compared to piperacillin when the same concentrations of both β-lactam standards were analyzed with TFME-LC-MS/MS. The spread of results (i.e., amplitude of fluctuations around the mean peak area of 96 replicates) observed for imipenem (Figure 1a) was slightly larger than the analogous spread of piperacillin peak areas (Figure 1b). This might be related to differences in the physicochemical properties of both compounds, determining their affinity to the divinylbenzene adsorbent. In this regard, the higher molecular weight, along with the higher hydrophobicity, of piperacillin compared to imipenem (MW_PIP_ = 517.56 [g/mol] vs. MW_IMI_ = 299.35 [g/mol], logP_PIP_ = 0.5 vs. logP_IMI_ = −3.9, respectively), results in stronger interactions with similarly hydrophobic divinylbenzene polimer (logP_DVB_ = 3.8). Nevertheless, all 96 replications (except one single point) of imipenem were within the range of the control limit (+/−2σ), testifying that there were no significant abnormalities in the manufacturing process and that the prepared TFME blades can be used for the precise analysis of β-lactams in this study.

### 2.2. TFME Procedure Optimization

To yield the optimal conditions for TFME extraction, the most important factors affecting its effectiveness were determined, including the selection of adsorbent material and an appropriate mixture of desorption solvents, time of adsorption and desorption. Parameters with the highest efficiency and the best precision (i.e., the lowest RSD) were chosen at each step of the optimization process.

#### 2.2.1. Solid Phase and Desorption Solvent Selection

Noticeably, the detector response for piperacillin was at least two orders of magnitude stronger than that for imipenem at the stage of solid-phase and desorption solvent optimization. As the analysis of piperacillin was not compromised, further attention was focused on the optimization of imipenem extraction.

**Figure 2:**
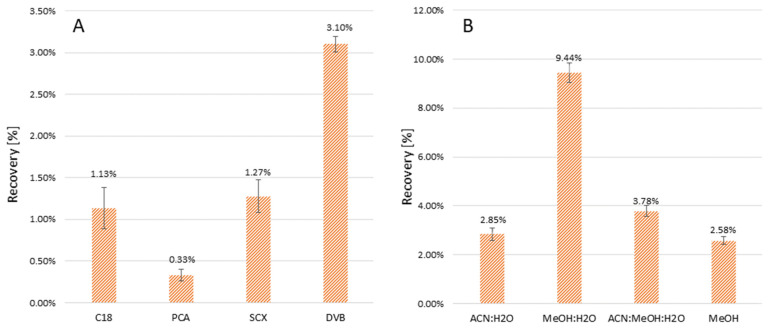
Optimization of imipenem recovery [%] for different adsorbents (**A**) and desorption solvents (**B**).

Four different adsorbents were tested—C18, PCA, SCX, DVB—amongst which the best extraction recovery (given as a ratio of analyte amount desorbed from TFME blade to analyte amount present in initial extraction solution) was observed for DVB as solid phase (Figure 2A). Amongst the four different tested desorption solvents—acetonitrile/water (1:1), methanol/water (1:1), acetonitrile/methanol/water (1:1:1) and pure methanol—the best results were obtained when a mixture of methanol:water (1:1) was used, yielding 9.44% imipenem recovery. 

While optimizing desorption parameters for TFME, one needs to consider the elution power, which cannot be greater for desorption solvent compared to the mobile phase used in LC. Otherwise, chromatographic separation will be altered towards reduced retention times, and analytes with low affinity to the stationary phase (such as imipenem in the applied settings) would be eluted in a dead retention time or would form more than one peak. Indeed, chromatographic separation was strongly impaired when a mixture of isopropanol:water (1:1) was used as a desorption solvent, resulting in the elution of imipenem within the dead retention time. For this reason, isopropanol:water (1:1) was not considered in the experiments presented here. For the methanol:water (1:1) mixture, analytes formed well shaped peaks, which were separate from each other.

#### 2.2.2. Extraction and Desorption Time

**Figure 3:**
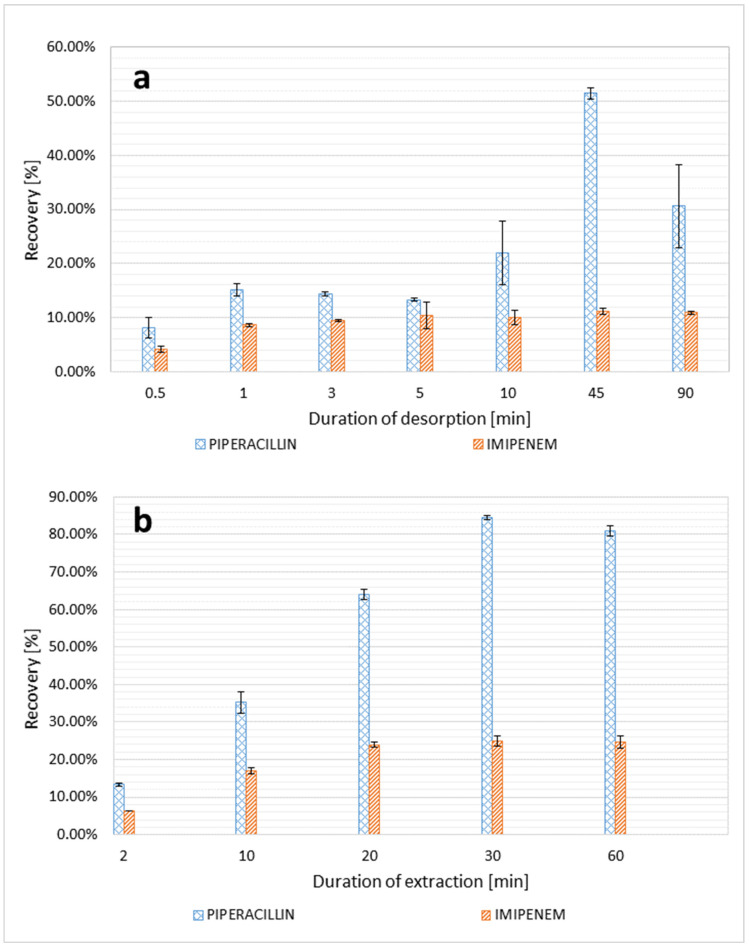
Recovery of piperacillin and imipenem for different durations of desorption (**a**) and extraction (**b**).

Since the extraction of the analyte from the sample to the TFME blade, as well as its later desorption to the solvent, only takes place until the equilibrium is reached between analyte concentrations in both phases (liquid–solid, solid–liquid), further extending the duration of both processes does not increase their efficiency. According to the results, the highest recovery and the best precision was achieved for a 30-min extraction time and 45-min desorption time (Figure 3).

### 2.3. Method Validation

#### 2.3.1. Linearity Range

**Figure 4:**
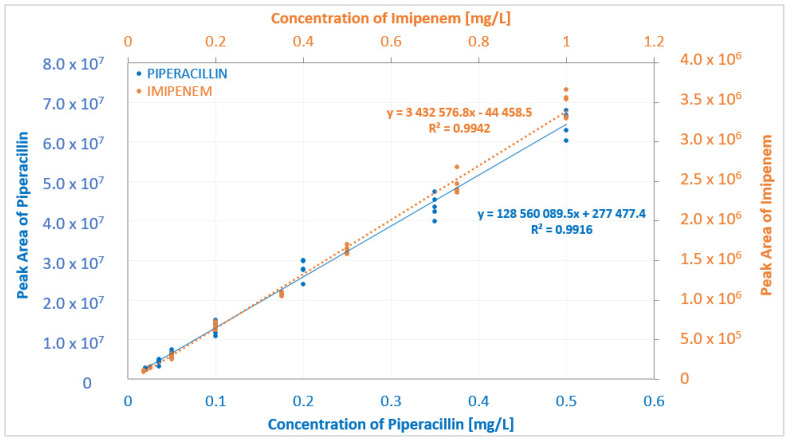
Calibration curves for extraction of imipenem and piperacillin from human blood plasma.

Respecting the FDA validation criteria given in Section 3.4.1., the linear regression coefficient (R2) for the calibration curve for piperacillin was 0.9916, and for imipenem it was 0.9934 (Figure 4). For imipenem, all back-calculated values were within the accepted ±15% (±20% for LLOQ) of the theoretical value. For piperacillin, a single measurement exceeded the ±15% accuracy that is allowed at four QC levels, but the calibration curve still met the acceptance criteria (≥75% of samples at each level).

#### 2.3.2. LLOQ and Sensitivity

The LLOQ obtained for piperacillin was 0.02 mg/L, with a mean accuracy and precision of 91.45% and 9.98%, respectively. For imipenem, the LLOQ was determined at 0.05 mg/L, with the mean accuracy and precision of 116.32% and 2.27%, respectively. The experimental results confirmed sufficient sensitivity for both analytes. The blank:analyte response at LLOQ ratio for imipenem was ≤16.95%, with a mean accuracy of 105.93% and precision of 16.08%, while for piperacillin it was ≤1.14%, with a mean accuracy of 91.14% and precision of 13.57%, for all of fifteen replicates (five in each of the three runs).

#### 2.3.3. Accuracy and Precision

Within- and between-run accuracy for both analytes were mostly within the accepted ranges (85–115% of nominal concentration), comprising 93% of samples for imipenem and 87% of samples for piperacillin. The precision met the acceptance criteria (RSD ≤15%) for all calibrators. The results for accuracy and precision are listed in Table 3 and Table 4, respectively.

#### 2.3.4. Selectivity and Specificity

No interferences were observed when analyzing the blank plasma samples from six ventilated patients who did not receive discussed antibiotics. Due to the complex composition of blood and BAL samples investigated in this study, the putative influence of biological matrix on the mass-spectrometric analysis of β-lactams was considered and evaluated according to the protocol described in Section 3.4.4. The matrix effect for imipenem and piperacillin at the same concentrations of 0.01, 0.1, 0.5 and 1 mg/L was found to be <4% (99.49–96.17% of nominal value for pure standards) and <6% (98.5–94.11% of its nominal value), respectively. These results confirm that the TFME method enables a reliable quantitative LC-MS/MS analysis of target analytes that are unaffected by matrix-related factors, even in very complex samples.

#### 2.3.5. Carryover

The peaks found in the blank sample (n = 4) injected right after the highest calibration standard were in the range of 1.26–4.13% of LLOQ for piperacillin, while for imipenem they were in the range of 0.37–2.10% of LLOQ. Therefore, both antibiotics passed the acceptance criteria of ±20% of LLOQ (Table 5).

#### 2.3.6. Recovery

The mean recoveries of TFME extraction were 59.01%, 56.13% and 73.54% for piperacillin prepared at concentrations of 0.06, 0.25 and 0.5 mg/L, respectively, while the mean recoveries for imipenem prepared at concentrations of 0.15, 0.5 and 1.0 mg/L were 26.10%, 19.05% and 22.83%, respectively. The results confirm that the proposed TFME-LC-MS/MS method meets the FDA Guidelines for Bioanalytical Method Validation.

### 2.4. Application in Biological Samples

#### 2.4.1. Patient Characteristics

Altogether, 14 paired blood samples were collected from 11 VAP patients receiving piperacillin, and another 13 paired blood samples were collected from 7 VAP patients receiving imipenem. The detailed characteristics of patients recruited for the determination of both antibiotics in plasma is given in Table 6.

Six paired BAL samples (as described in Section 3.5.) were collected from six VAP patients receiving piperacillin and another five paired BAL samples were collected from three VAP patients receiving imipenem. The detailed characteristics of patients recruited for the determination of both antibiotics in bronchoalveolar lavage is given in Table 7.

#### 2.4.2. Concentration of Piperacillin and Imipenem in Plasma

The first group considered consists of patients receiving 4 g of piperacillin as an intravenous infusion over 20 min in 6-h intervals. According to the Summary of Product Characteristics (SmPC) for Piperacillin/Tazobactam (4 g/0.5 g), the maximum concentration of piperacillin after infusion in healthy volunteers was 298 µg/mL [35]. The results obtained demonstrate that, for some of the critically ill patients enrolled in this study, the post-infusion concentration of piperacillin in plasma was higher than the declared literature value for healthy subjects. Moreover, for a few subjects, an elevated plasma level was also found before drug infusion (Table 8). It should, however, be noted that VAP patients enrolled in this study were receiving antibiotics over different periods, usually several days before the collection of blood/BAL specimen, which may lead to the accumulation of a drug in the body, thus affecting the intra- and inter-patient variations in antibiotic concentrations measured with TFME-LC-MS/MS. This is in line with other studies, where high piperacillin variabilities were reported in the critically ill [11].

In patients treated with imipenem, 1 g of this antibiotic was administered every 8 h as an infusion over 3 h. According to the Summary of Product Characteristics (SmPC) for Imipenem/Cilastatin, the maximal imipenem concentration in plasma after infusion of 1 g in healthy volunteers was in the range of 41–83 µg/Ll, reaching the mean maximal value of 66 µg/mL [36]. The observed concentrations of imipenem in plasma 1 h after infusion were below the SmPC values, ranging from 5.8 to 38.8 mg/L (Table 8).

**Figure 5:**
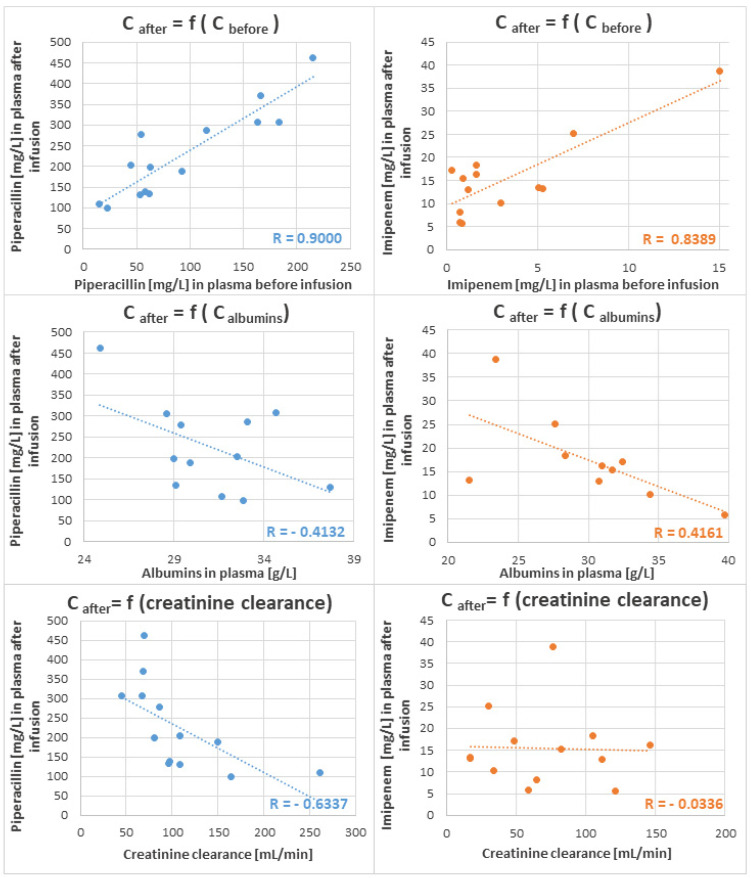
Concentrations [mg/L] of piperacillin (blue, left panel) and imipenem (orange, right panel) in plasma samples collected 1 h after the end of intravenous infusion in the function of antibiotic base-level before infusion (upper panel), albumins [g/L] in plasma (middle panel) and creatinine [mmol/L] in plasma (bottom panel). Tentative linear regression is given as a dotted line along with the respective value of the Pearson Correlation Coefficient.

A positive correlation between concentrations of both antibiotics in plasma before and after infusion was found (Figure 5), whereby the Pearson Correlation Coefficient reached the value of r = 0.9000 for piperacillin and r = 0.8389 for imipenem. A negative tendency between the concentrations of unbound β-lactams in plasma after infusion and albumin level in blood was observed for piperacillin and imipenem; nevertheless, the Pearson Correlation Coefficients exhibited relatively low values (r = −0.6617 and r = −0.4132, respectively), indicating a weak relationship between these variables. A similar observation was reported by Wong et al. [13], who also could not find a significant dependence between albumin concentrations and unbound β-lactams (except for flucloxacillin). This may be linked with a nonlinearity of protein binding, which considerably hinders the prediction of available unbound antibiotics in the blood. Additionally, a negative dependence was observed between plasma piperacillin and creatinine clearance, with a similarly low value of the Pearson Correlation Coefficient (r = −0.6337), which may be related to the small number of patients enrolled in our study. Intriguingly, no dependence at all was observed between imipenem in plasma and creatinine clearance.

#### 2.4.3. Degree of Penetration of Piperacillin and Imipenem to BAL

In the case of piperacillin, the experimentally determined degree of penetration to BAL was in the range 1.88–2.71% (Table 9). According to the research of Felton et al. [9], the average degree of penetration of piperacillin into the pulmonary epithelial lining fluid was 43.9%; however, the values determined in their study were in the very wide range of 2.0–515.9%. This demonstrates the problem with the unpredictability of piperacillin penetration into the lungs and shows that the optimal dosing of piperacillin in the critically ill is a true challenge. The low penetration of piperacillin into the epithelial lining fluid may lead to sub-therapeutic concentrations; hence, the current treatment regimens may be ineffective in patients suffering from ventilation-associated pneumonia. Similarly, the distribution of imipenem in critically ill patients is much smaller compared with the respective distribution values in healthy individuals. In this regard, Tegeder et al. [10] have shown that the maximum concentration of unbound imipenem in infected organs in critically ill patients was only 5% of the maximum concentration achieved in plasma, due to its weak perfusion into internal organs. The penetration of unbound imipenem into BAL determined in the presented study (Table 9) ranged between 1.49 and 11.15% of its plasma levels. It should also be mentioned that mechanically ventilated patients suffering from VAP were enrolled in this study, in whom lung perfusion could be unstable and variable during hospitalization.

**Figure 6:**
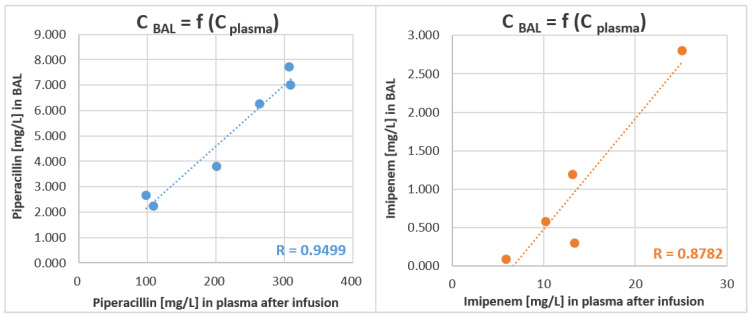
Concentrations [mg/L] of piperacillin (blue, left panel) and imipenem (orange, right panel) in BAL samples in the function of antibiotic level in plasma [mg/L]. Both sample types were collected at the same time, i.e., one hour after the end of intravenous infusion. Tentative linear regression is given as a dotted line along with the corresponding value of the Pearson Correlation Coefficient.

It should be stated that in the presented study both BAL and blood samples were collected from each patient at the same time (i.e., 1 h after infusion); hence, sampling bias was eliminated and could not deteriorate the comparison of antibiotic concentrations in both matrices. Indeed, good relationship was observed between drug concentration in BAL and plasma (Figure 6). Noticeably, the applied TFME technique only extracts the free fraction of drugs, i.e., those that are unbound to the plasma proteins; hence, the final LC-MS/MS analysis directly determines the biologically active part of antibiotics. This means that the concentrations were not recalculated from the total drug concentration using published protein binding values, which were proved by other researchers to significantly differ in healthy volunteers and critically ill patients [13]. This fact certainly contributes to the differences in blood/BAL partition observed here and in other studies.

## 3. Materials and Methods

### 3.1. Chemicals and Reagents

Imipenem (IMI) and sodium salt of piperacillin (PIP) were used as the standards, N,N-dimethylformamide (DMF) and polyacrylonitrile (PAN) were purchased from Merck KGaA (Darmstadt, Germany). PCA Chromabond^®^, C18 Chromabond^®^, Easy Chromabond^®^ (DVB) and SA Chromabond^®^(SCX) were supplied from PAS Technology (Magdala, Germany). Hydrochloric acid (HCl) was purchased from Chempur^®^ (Piekary Śląskie, Poland). LC-MS/MS grade methanol (MeOH), acetonitrile (ACN) and formic acid (FA) were supplied from Honeywell|Fluka™ (Seelze, Germany). LC-MS/MS grade isopropanol (i-PrOH) was purchased from Honeywell|Riedel de Haen™ (Seelze, Germany) and LiChrosolv^®^ water was supplied from Merck KGaA (Darmstadt, Germany).

### 3.2. Instrumentation

All analyses were performed using Shimadzu LC-MS/MS 8060 system, equipped with autosampler Nexera X2 SIL-30AC and two pumps Nexera X2 LC-30AD (all from Shim-Pol, Warsaw, Poland).

#### 3.2.1. Optimization of LC-MS Conditions

The optimization of LC-MS conditions was performed by the direct injection of standard solution in concentration 0.1 µg/mL. Chromatographic separation conditions were established by comparing three chromatographic columns: Kinetex^®^ 1.7 µm F5 100 Å 100 mm × 2.1 mm (Phenomenex, Torrance, CA, USA), Kinetex^®^ 2.6 µm C18 100 Å 100 mm × 3 mm (Phenomenex, Torrance, CA, USA) and Acquity UPLC^®^ BEH C18 1.7 µm 130 Å 100 mm × 2.1 mm (Waters, Milford, MA, USA) and five different mobile phase gradients. The optimization of MS conditions was focused on the flow rates and temperatures of gases as well as the selection of parent and product ions *m*/*z* for each analyte. It also comprised voltage optimization of the Q1 and Q3 and collision energy for each multiple-reaction-monitoring (MRM) transition.

#### 3.2.2. Final LC-MS Conditions

Gradient elution of water with 0.1% FA (mobile phase A) and ACN with 0.1% FA (mobile phase B) was used for chromatographic separation with a summary flowrate of 0.4 mL/min and a total run time of 7.2 min. The column temperature was kept at 40 °C, while the injection volume was 10 µL. The ion source temperature was set at 150 °C and positive electro-spray ionization (ESI+) was used. The following parameters were applied: nebulizing gas flow at 3 L/min, heating gas flow at 10 L/min, drying gas flow at 5 L/min, interface temperature at 300 °C and heat block temperature at 400 °C.

#### 3.2.3. Preparation of TFME Coating

Before the coating process, the blades were sonicated in HCl (purity of 36%) for 60 min to clean and activate a stainless-steel surface for effective coverage with adsorbent. Afterwards, blades were rinsed with distilled water, dried at 150 °C for 30 min and cooled to room temperature. Four types of adsorbents were used: C18, DVB, SCX and PCA, which were immobilized on blades surface with biocompatible PAN glue. The PAN glue was prepared for each adsorbent by mixing 1g of PAN and 14.47 mL of DMF, heating at 90 °C for about 1 h and cooling to room temperature. Then, the chosen adsorbent particles (2.375 g) were added to PAN-DMF mixture and coating was performed by spraying with a flask-type sprayer connected to nitrogen (as carrier gas). The thin layers of adsorbent-PAN slurry were deposited on app. 1.5 cm of blade-end, followed by instant thermal curing in the oven. Conditions for each sorbent are presented in Table 10. Afterwards, all adsorbent coatings immobilized on blades were equalized to 1 cm.

#### 3.2.4. Quality Control of TFME Blades

To control the quality of the prepared brushes, the TFME sampling procedure was used for extraction of mixed piperacillin and imipenem at a concentration 0.1 µg/mL in phosphate-buffered saline (PBS). Shewhart charts were made based on integrated peak areas for respective analytes.

### 3.3. Optimization of TFME Procedure

A standard solution of mixed imipenem and piperacillin at concentration 0.1 mg/L was used to optimize TFME procedure. To achieve maximum extraction efficiency, four different adsorbents were investigated: C18, PCA, SCX and DVB. Since the desorption solvent itself can exhibit high-power elution, altering chromatographic separation, four desorption solutions were considered: can/water (1:1), MeOH/water (1:1), ACN/MeOH/water (1:1:1) and pure MeOH. Finally, five different extraction times (5, 15, 30, 60 and 90 min) and seven different desorption times (0.5, 1, 3, 5, 15, 45, 90 min) were compared for the optimization of TFME procedure.

### 3.4. Method Validation

The method was validated according to the guidelines of the US Food and Drug Administration (FDA) in bioanalytical method validation from 2018 [29].

#### 3.4.1. Linearity Range

The final concentrations of standards used for plotting calibration curves in human plasma were: 0.01, 0.02, 0.035, 0.05, 0.1, 0.2, 0.35, 0.5, 0.75 and 1 mg/L. The acceptance criteria were 85–115% of nominal concentration (except the lower limit of quantitation (LLOQ) with 80–120%) for at least six non-zero calibrator levels over 75% of all samples in each validation run. The slope, intercept, and correlation coefficient for both curves were determined using linear regression analysis.

#### 3.4.2. LLOQ and Sensitivity

Sensitivity was defined as the lowest non-zero standard on the calibration curve (LLOQ). Determined from the calibration curves, LLOQ samples were analyzed in five replicates in three separate runs. The acceptance criteria were: analyte response at LLOQ ≥ five times the analyte response of the blank, accuracy at ±20% of nominal concentration and precision ≤20% (CV%).

#### 3.4.3. Accuracy and Precision

Within-run and between-run accuracy and precision were determined at four concentration levels, using the four quality control (QC) samples: LLOQ, low (L, three times LLOQ), medium (M) and high (H), each in five replicates in three separated runs. Accuracy was given as the percentage ratio of test results to the expected values and precision was defined as the coefficient of variation (CV%). The acceptance criteria for within- and between-run accuracy were 85–115% of nominal concentration (except LLOQ, with 80–120% of nominal concentration) and, for precision, ≤15% (except LLOQ, with ≤20%). For both, 75% of samples had to meet the above criteria.

#### 3.4.4. Selectivity and Specificity

Selectivity was ensured by application of the multiple-reaction monitoring mode in MS acquisition, in addition to the confirmation of retention time. In this mode, a specific ion (precursor) selected in the first-stage quadrupole (Q1) was dissociated in collision cell yielding a selective and specific product ion detected after passing the second stage quadrupole (Q3).

Specificity, reflecting any potential influence of concomitant sample constituents (e.g., medications) on the determination of target antibiotics, including ion suppression/enhancement and extraction efficiency, was demonstrated by analyzing plasma samples from six ventilated patients without piperacillin or imipenem treatment. The acceptance criteria were noninterference at the retention times of analytes.

To assess the matrix effect, the detector response to the respective concentration of an analyte (QC standards: 0.01, 0.1 and 0.5 mg/L) spiked with a pure desorption solvent was compared to the same analytes’ concentrations when spiked to a desorption solvent after previous TFME extraction from blank plasma (derived from patients free of considered antibiotics).

#### 3.4.5. Carry-Over

Carry-over was assessed by the injection of blank samples right after the highest calibration standard. Signal for analytes in the blank samples should not exceed 20% of LLOQ.

#### 3.4.6. Recovery

For the determination of recovery, TFME-extracted samples at three QC levels (L, M and H) were compared to TFME-extracts of blanks spiked with the analyte post-extraction (at L, M and H). The acceptance criteria were: ≥67% of all QCs in ±15% of theoretical value and at least 50% of QCs per level in the range of ±15% of the theoretical value. The acceptance criterium for carry-over was a value ≤20% of LLOQ.

### 3.5. Blood and BAL Sampling from VAP Patients

All blood and BAL samples were collected from mechanically ventilated patients hospitalized in the Anesthesiology and Intensive Care Unit of the 10th Military Research Hospital and Polyclinic in Bydgoszcz. The study was supported by Polish National Science Centre (Grant No. 2017/26/D/NZ6/00136) and the agreement of the local Bioethics Committee was obtained under the number KB-218/2018. For the purpose of method validation, blood and BAL samples were taken from MV patients without imipenem and piperacillin treatment, but with no restrictions regarding other medications.

Before applying the validated method in clinical settings, the patients enrolled in this study had to fulfill the following inclusion criteria: (1) admission to ICU, (2) mechanical ventilation, (3) suspicion or confirmation of VAP (based on clinical and microbiological evidence), (4) ongoing imipenem or piperacillin treatment. The exclusion criteria were: (1) age under 18, (2) pregnancy, (3) existing lung disease (traumatic lung injury or pulmonary cancer), (4) strict isolation at ICU, (5) increased intracranial pressure (ICP), (6) positive end-expiratory pressure (PEEP) >10, (6) extra corporal heart and lung assistance devices.

For patients treated with imipenem or piperacillin, blood samples were collected just before starting the infusion and 1 h after the end of infusion. Blood samples were collected to sodium citrate tubes and centrifuged for 6 min at 3000 rpm. Plasma samples were stored at −80 °C until analysis. Under the applied settings, a “blood sample” should be understood as a pair consisting of pre- and post-infusion blood.

BAL samples were taken as part of a procedure for the clearance of excessive fluid from the patient’s respiratory tract. Since this action was done approximately 1 h after the end of antibiotic infusion, the obtained samples could be used to estimate a degree of drug penetration into the pulmonary epithelial lining fluid. Under the applied settings, a “BAL sample” should be understood as a pair consisting of plasma and BAL collected around 1 h after the end of drug infusion.

Prior to the TFME procedure, all plasma samples collected from MV patients receiving piperacillin were diluted 10,000-fold, while BAL samples were diluted 1000-fold with PBS. Samples from MV patients treated with imipenem were diluted 100-fold for plasma collected after infusion, while 10-fold dilution was used for BAL and plasma samples collected before infusion. For all types of samples, the TFME procedure, optimized and validated according to the protocol described above, was applied.

## 4. Study Limitations

Given the invasiveness of the BAL sampling procedure, the collection of a single specimen per patient (instead of multiple timepoints) can be considered a limitation of our work. The small number of patients enrolled in this study, along with the variability of clinical conditions influencing the distribution of beta-lactam and their PK in the critically ill state, provide another limitation. Additionally, the fact that biological material was collected from VAP patients at different periods of the enrolled antimicrobial therapy, could contribute to the study limitations, hindering the comparison of the obtained results and the literature values. Nevertheless, this work aimed towards the development of a new analytical tool based on the TFME extraction technique and a demonstration of its usefulness in the determination of active unbound beta-lactams in biological material, not for the investigation of therapy outcomes.

## 5. Conclusions

The validation results confirm that this method utilizing TFME-LC-MS/MS is suitable for the selective, sensitive, accurate and precise analysis of chosen beta-lactams in both plasma and BAL samples. The simultaneous high-throughput analysis of as many as 96 samples is possible, substantially reducing the time and cost of analysis. The application of TFME extraction ensures not only a very low matrix effect, enabling the highly specific determination of analytes in complex biological material, but also the direct analysis of a sole unbound fraction of antibiotics. The validated method was successfully applied to determine the penetration of piperacillin and imipenem into BAL fluid, demonstrating that measured unbound concentrations of both antibiotics substantially differ from the values estimated by recalculation from total concentrations using published protein-binding data. Additional evidence for substantial variability in nonlinear plasma-protein binding, dependence on creatinine clearance, and poor permeability to the site of infection may lead to inadequate subtherapeutic concentrations of piperacillin and, to some extent, to imipenem. Therefore, the presented study provides a valuable analytical tool offering a step towards personalized therapeutic drug monitoring.

## Figures and Tables

**Table 1:** Gradient elution program—mobile phase composition.

Time (min)	% A Phase	% B Phase
0.01	95.00	5.00
0.20	95.00	5.00
1.00	60.00	40.00
2.00	60.00	40.00
3.50	5.00	95.00
5.00	5.00	95.00
5.01	95.00	5.00
7.20	95.00	5.00

**Table 2:** LC-MS/MS parameters for imipenem and piperacillin.

Compounds	Parent Ion *m*/*z*	Product Ions *m*/*z*	Collision Energy (V)	Q1 (V)	Q3 (V)	Retention Time (min)
Imipenem	300.00	141.85	−28.00	−10.00	−14.00	1.76
		98.05	−28.00	−10.00	−19.00	
		125.95	−20.00	−13.00	−19.00	
Piperacillin	518.05	143.00	−20.00	−22.00	−15.00	2.53
		159.95	−19.00	−22.00	−30.00	
		359.00	−10.00	−22.00	−12.00	

**Table 3:** Within- and between-run accuracy presented as the percent of nominal concentration of the analyte.

Compounds	Concentration [mg/L]	Within-Run [%]			Between-Run [%]
		1st Run	2nd Run	3rd Run	
Imipenem	0.05	110.54–115.53	109.07–112.82	88.52–92.87	88.52–115.53
	0.15	102.02–112.09	99.88–106.51	88.60–108.93	88.60–112.09
	0.5	85.19–98.33	71.47–92.74	94.83–102.00	71.47–102.00
	1.0	91.68–100.12	93.65–86.91	97.33–107.89	86.91–107.89
Piperacillin	0.02	83.26–118.56	83.93–113.37	78.14–100.68	79.14–118.56
	0.06	88.25–111.57	90.36–111.57	81.82–116.08	81.82–116.08
	0.25	96.13–117.56	88.65–107.25	86.49–102.08	88.65–117.56
	0.5	95.15–103.52	85.08–103.78	92.68–99.15	85.08–103.78

**Table 4:** Within- and between-run precision presented as the Relative Standard Deviation [%].

Compounds	Concentration [mg/L]	Within-Run [%]			Between-Run [%]
		1st Run	2nd Run	3rd Run	
Imipenem	0.05	1.81	1.35	2.06	10.59
	0.15	3.67	2.77	9.05	6.91
	0.5	5.90	10.49	2.79	11.48
	1.0	3.38	2.72	4.54	6.47
Piperacillin	0.02	14.73	13.12	11.51	13.43
	0.06	10.43	9.32	10.38	10.72
	0.25	9.29	8.09	7.64	9.74
	0.5	3.38	7.81	2.87	5.08

**Table 5:** Carryover presented as the ratio of Peak Area for blank and Peak Area for LLOQ.

Compounds	Peak Area for Blank	Peak Area for LLOQ	% of LLOQ
Imipenem	4.40 × 10^3^	2.09 × 10^5^	2.10%
	7.73 × 10^2^		0.37%
	3.24 × 10^3^		1.55%
	4.05 × 10^3^		1.94%
Piperacillin	2.82 × 10^4^	6.84 × 10^5^	4.13%
	8.61 × 10^3^		1.26%
	1.56 × 10^4^		2.28%
	2.77 × 10^4^		4.05%

**Table 6:** Characteristics of patients included in blood sampling. Other antibiotics are considered only if administrated within one week before sample collection. PCT—procalcitonin reference range: <0.05 ng/mL; CRP—C-reactive protein reference range: <5 mg/L; Albumins reference range: 32–48 g/L; Urea reference range: 3.2–8.2 mmol/L; Creatinine reference range: 61.9–114.9 µmol/L; CVVHD—continuous veno-venous hemodialysis; FSD—furosemide.

Pat. #	Age [years]	Body Weight [kg]	Microorganisms Found	Other Drugs	Renal Impairment Therapy	PCT [ng/mL]	CRP [mg/L]	Albumins [g/L]	Urea [mmol/L]	Creatinine [µmol/L]
Patients receiving PIPERACILLIN										
1	89	88	*P. aeruginosa, P. mirabilis, Enterococcus* spp.	-	-	1.27	88.69	34.68	9.4	82.06
2	28	78	*P. aeruginosa, S. marcescens*	-	-	0.13	35.66	33.1	11.6	29.36
3	60	71	*P. mirabilis, E. coli*	-	-	1.4	69.56	29.1	5.3	61.71
				-		0.69	51.08	-	4.3	61.2
				-		0.39	53.65	32.54	3.6	54.36
4	73	64	*-*	-	-	0.59	76.48	29.37	8.6	50.87
						0.45	42.08	28.99	9.3	55.67
5	83	82	*-*	-	-	0.3	133.56	24.9	13.5	82.31
6	66	86	*P. mirabilis, E. faecalis*	-	CVVHD	0.67	179.61	-	10.3	96.98
7	64	88	*E. coli*	-	-	0.19	109.19	29.89	4.1	54.7
8	69	86	*E. coli, Candida* spp.	-	-	0.06	60.14	31.64	4	28.72
9	68	68	*S. hominis*	-	-	0.15	6.26	37.67	3	46.89
10	46	76	*K. pneumoniae*	-	-	0.28	184.35	32.86	10.1	53.53
11	77	62	*C. albicans*	-	-	2.37	195.97	28.58	13.6	105.33
Patients receiving IMIPENEM										
1	71	83	*E.coli*	piperacillin	diuresis supported with FSD	24.39	105.11	31.73	11	72.62
						2.88	26.4	30.95	6.4	40.82
2	57	75	*P. mirabilis*	piperacillin	CVVHD	10.04	241.85	-	12.1	118.62
						5.2	297.15	32.45	11.6	111.15
3	77	91	*E.coli, C. albicans*	-	diuresis supported with FSD	2.09	231.29	28.33	4.8	66.99
						1.21	140.11	-	5.1	58.17
						1.1	124.79	30.78	6.9	63.08
4	59	84	*S. marcescens, S. aureus*	-	-	30.2	78.93	23.4	18.3	109.19
5	71	78	*S. aureus*	ceftriaxone	CVVHD	4.72	369.99	27.64	11.1	220.28
						6.24	366.4	34.44	16	196.23
6	92	68	*K. pneumoniae*	piperacillin	diuresis supported with FSD	2.64	377.4	-	23.3	198.76
						2.47	324.75	21.52	27	197.61
7	30	78	*Candida* spp.	Piperacillin	diuresis supported with FSD	2.36	327.57	39.73	14.4	178.12

**Table 7:** Characteristics of VAP patients included in BAL sampling. Other antibiotics are considered only if administrated within one week before sample collection. week before imipenem administration; PCT—procalcitonin reference range: < 0.05 ng/mL; CRP—C-reactive protein reference range: <5 mg/L; Albumins reference range: 32–48 g/L; Urea reference range: 3.2–8.2 mmol/L; Creatinine reference range: 61.9–114.9 µmol/L; CVVHD—continuous venovenous hemodialysis; FSD—furosemide.

Pat. #	Age [years]	Body Weight [kg]	Microorganisms Found	Other Drugs	Renal Impairment Therapy	PCT [ng/mL]	CRP [mg/L]	Albumins [g/L]	Urea [mmol/L]	Creatinine [µmol/L]
Patients receiving PIPERACILLIN										
1	66	86	*P. mirabilis, E. faecalis*	-	CVVHD	0.71	263	31.57	16	164
2	28	76	*P. aeruginosa, S. marcescens*	-	-	0.13	35.66	33.1	11.6	29.36
3	68	84	*C. freundi*	-	-	37.96	61	22.56	9.1	117
4	69	78	*E. coli, Candida* sp.	-	-	0.06	60.14	31.64	4	28.72
5	46	67	*K. pneumoniae*	-	-	0.28	184.35	32.86	10.1	53.53
6	77	79	*C. albicans*	-	-	2.37	195.97	28.58	13.6	105.33
Patients receiving IMIPENEM										
1	71	78	*S. aureus*	ceftriaxone	CVVHD	4.72	369.99	27.64	11.1	220.28
						6.24	366.4	34.44	16	196.23
2	92	68	*K. pneumoniae*	piperacillin	diuresis supported with FSD	2.64	377.4	-	23.3	198.76
						2.47	324.75	21.52	27	197.61
3	30	78	*Candida* spp.	piperacillin	diuresis supported with FSD	2.36	327.57	39.73	14.4	178.12

**Table 8:** Plasma concentration of unbound piperacillin and unbound imipenem before and after drug administration according to the protocol described in the text. The value “0” (zero) in antibiotic treatment duration indicates that the day of sample collection is the first day of receiving the respective antibiotic.

Pat. #	Sample Number	Antibiotic Treatment before Sampling [days]	Concentration in Plasma before Infusion [mg/L]	Concentration in Plasma after Infusion [mg/L]
Patients receiving PIPERACILLIN				
1	PIP-1	1	162.869	307.806
2	PIP-2	7	115.500	286.148
3	PIP-3	3	61.603	133.925
	PIP-4	4	58.241	138.191
	PIP-5	5	44.869	203.747
4	PIP-6	6	54.496	278.231
	PIP-7	7	62.556	198.964
5	PIP-8	1	215.263	461.537
6	PIP-9	1	166.428	370.821
7	PIP-10	3	92.074	189.133
8	PIP-11	0	14.533	109.166
9	PIP-12	2	53.270	130.710
10	PIP-13	1	22.617	98.457
11	PIP-14	2	183.280	307.027
Patients receiving IMIPENEM				
1	IMI-1	2	0.918	15.344
	IMI-2	4	1.646	16.283
2	IMI-3	4	0.756	8.176
	IMI-4	5	0.293	17.185
3	IMI-5	4	1.650	18.394
	IMI-6	5	0.835	5.627
	IMI-7	6	1.157	12.919
4	IMI-8	n.a.	15.034	38.816
5	IMI-9	0	7.001	25.113
	IMI-10	3	2.958	10.219
6	IMI-11	1	5.067	13.399
	IMI-12	2	5.291	13.164
7	IMI-13	0	0.707	5.852

**Table 9:** The degree of penetration of unbound piperacillin and unbound imipenem to BAL.

Pat. #	Sample Number	Concentration after Infusion in PLASMA [mg/L]	Concentration after Infusion in BAL [mg/L]	BAL/Plasma %
Patients receiving PIPERACILLIN				
1	PIP-I	201.687	3.794	1.88
2	PIP-II	263.928	6.271	2.38
3	PIP-III	309.428	6.995	2.26
4	PIP-IV	109.166	2.236	2.05
5	PIP-V	98.457	2.673	2.71
6	PIP-VI	307.027	7.711	2.51
Patients receiving IMIPENEM				
1	IMI-I	25.113	2.801	11.15
2	IMI-II	10.219	0.576	5.64
	IMI-III	13.399	0.298	2.22
3	IMI-IV	13.164	1.189	9.03
	IMI-V	5.852	0.087	1.49

**Table 10:** Conditions of sorbent application depending on the type of sorbent.

Type of Sorbent	Number of Layers	Oven Temperature (°C)	Curing Time (min)
C18	10	180 °C	2 min
DVB	8	110 °C	3 min
PCA	10	110 °C	3 min
SCX	10	110 °C	3 min

## Data Availability

Not applicable.

## Sample Availability

Samples of the biological material from VAP patients are not available from the authors.

## References

[1] Mirtalaei N., Farazi A., Ebrahimi Monfared M., Jokar A. (2019). Efficacy of antibiotic prophylaxis against ventilator-associated pneumonia. J. Hosp. Infect..

[2] Park D.R. (2005). Microbiology of Ventilator-Associated Pneumonia. Respir. Care.

[3] Joseph N.M., Sistla S., Dutta T.K., Badhe A.S., Rasitha D., Parija S.C. (2010). Ventilator-associated pneumonia in a tertiary care hospital in India: Role of multi-drug resistant pathogens. J. Infect. Dev. Ctries..

[4] Karaoglan H., Yalcin A.N., Cengiz M., Ramazanoglu A., Ogunc D., Erbay R.H., Yilmaz M., Mamikoglu L. (2010). Cost analysis of ventilator-associated pneumonia in Turkish medical-surgical intensive care units. Infez. Med..

[5] Roberts J.A., Lipman J. (2009). Pharmacokinetic issues for antibiotics in the critically ill patient. Crit. Care Med..

[6] Roberts J.A., Hons B.P., Kruger P., Paterson D.L., Lipman J. (2008). Antibiotic resistance—What’s dosing got to do with it?. Crit. Care Med..

[7] Chastre J., Wunderink R., Prokocimer P., Lee M., Kaniga K., Friedland I. (2008). Efficacy and safety of intravenous infusion of doripenem versus imipenem in ventilator-associated pneumonia: A multicenter, randomized study. Crit. Care Med..

[8] Hayashi Y., Roberts J.A., Paterson D.L., Lipman J. (2010). Pharmacokinetic evaluation of piperacillin-tazobactam. Expert Opin. Drug Metab. Toxicol..

[9] Felton T.W., McCalman K., Malagon I., Isalska B., Whalley S., Goodwin J., Bentley A.M., Hope W.W. (2014). Pulmonary penetration of piperacillin and tazobactam in critically ill patients. Clin. Pharmacol. Ther..

[10] Tegeder I., Schmidtko A., Bräutigam L., Kirschbaum A., Geisslinger G., Lötsch J. (2002). Tissue distribution of imipenem in critically ill patients. Clin. Pharmacol. Ther..

[11] Zander J., Döbbeler G., Nagel D., Scharf C., Huseyn-Zada M., Jung J., Frey L., Vogeser M., Zoller M. (2016). Variability of piperacillin concentrations in relation to tazobactam concentrations in critically ill patients. Int. J. Antimicrob. Agents.

[12] Karampitsakos T., Papaioannou O., Kaponi M., Kozanidou A., Hillas G., Stavropoulou E., Bouros D., Dimakou K. (2020). Low penetrance of antibiotics in the epithelial lining fluid. The role of inhaled antibiotics in patients with bronchiectasis. Pulm. Pharmacol. Ther..

[13] Wong G., Briscoe S., Adnan S., McWhinney B., Ungerer J., Lipman J., Roberts J.A. (2013). Protein binding of β-lactam antibiotics in critically Ill patients: Can we successfully predict unbound concentrations?. Antimicrob. Agents Chemother..

[14] Hites M., Taccone F.S., Wolff F., Cotton F., Beumier M., De Backer D., Roisin S., Lorent S., Surin R., Seyler L. (2013). Case-control study of drug monitoring of β-lactams in obese critically ill patients. Antimicrob. Agents Chemother..

[15] Neuner E.A., Ahrens C.L., Groszek J.J., Isada C., Vogelbaum M.A., Fissell W.H., Bhimraj A. (2012). Use of therapeutic drug monitoring to treat Elizabethkingia meningoseptica meningitis and bacteraemia in an adult. J. Antimicrob. Chemother..

[16] Roberts J.A., Ulldemolins M., Roberts M.S., Mcwhinney B., Ungerer J., Paterson D.L., Lipman J. (2010). International Journal of Antimicrobial Agents Therapeutic drug monitoring of β-lactams in critically ill patients: Proof of concept. Int. J. Antimicrob. Agents.

[17] Petrosillo N., Giannella M., Lewis R., Viale P., Petrosillo N. (2014). Treatment of carbapenem-resistant Klebsiella pneumoniae: The state of the art. Expert Rev. Anti-Infect. Therapy.

[18] Charmillon A., Novy E., Agrinier N., Leone M., Kimmoun A., Levy B., Demoré B., Dellamonica J., Pulcini C. (2016). The ANTIBIOPERF study: A nationwide cross-sectional survey about practices for β-lactam administration and therapeutic drug monitoring among critically ill patients in France. Clin. Microbiol. Infect..

[19] Denooz R., Charlier C. (2008). Simultaneous determination of five β-lactam antibiotics (cefepim, ceftazidim, cefuroxim, meropenem and piperacillin) in human plasma by high-performance liquid chromatography with ultraviolet detection. J. Chromatogr. B.

[20] Verdier M.-C., Tribut O., Tattevin P., Le Tulzo Y., Michelet C. (2011). Simultaneous Determination of 12 β-Lactam Antibiotics in Human Plasma by High-Performance Liquid Chromatography with UV Detection: Application to Therapeutic Drug Monitoring. Antimicrob. Agents Chemother..

[21] Wolff F., Deprez G., Seyler L., Taccone F., Hites M., Gulbis B., Vincent J.-L., Jacobs F., Cotton F. (2013). Talanta Rapid quantification of six b -lactams to optimize dosage regimens in severely septic patients. N. Engl. J. Med..

[22] Gu G., Black M., Cookson C., Fiorella A., Li Y., Gorman S.H., Bakhtiar R. (2018). Validation of an LC-MS/MS method for simultaneous quantification of venlafaxine and its five metabolites in rat plasma and its application in a pharmacokinetic study. J. Chromatogr. B.

[23] Naicker S., Valero Y.C.G., Meija J.L.O., Lipman J., Roberts J.A., Ord L., Wallis C., Parker S.L. (2017). A UHPLC-MS/MS method for the simultaneous determination of piperacillin and tazobactam in plasma (total and unbound), urine and renal replacement therapy effluent. J. Pharm. Biomed. Anal..

[24] El-Najjar N., Jantsch J., Gessner A. (2017). The use of liquid chromatography-tandem mass spectrometry for therapeutic drug monitoring of antibiotics in cancer patients. Clin. Chem. Lab. Med..

[25] Barco S., Bandettini R., Maffia A., Tripodi G., Cangemi G. (2015). Quantification of piperacillin, tazobactam, meropenem, ceftazidime, and linezolid in human plasma by liquid chromatography/tandem mass spectrometry. J. Chemother..

[26] Tsai I., Sun H., Chen G., Lin S., Kuo C. (2013). Talanta Simultaneous quantification of antimicrobial agents for multidrug-resistant bacterial infections in human plasma by ultra-high-pressure liquid chromatography-tandem mass spectrometry. Talanta.

[27] Pawliszyn J. (2012). Handbook of Solid Phase Microextraction.

[28] Filipiak W., Bojko B. (2019). SPME in clinical, pharmaceutical, and biotechnological research-how far are we from daily practice?. TrAC Trends Anal. Chem..

[29] US Food and Drug Administration Bioanalytical Method Validation Guidance for Industry. https://www.fda.gov/files/drugs/published/Bioanalytical-Method-Validation-Guidance-for-Industry.pdf..

[30] Mabilat C., Gros M.F., Nicolau D., Mouton J.W., Textoris J., Roberts J.A., Cotta M.O., Van Belkum A., Caniaux I. (2019). Diagnostic and medical needs for therapeutic drug monitoring of antibiotics. Eur. J. Clin. Microbiol Infect. Dis.

[31] Legrand T., Vodovar D., Tournier N., Khoudour N. (2016). Simultaneous Determination of Eight β-Lactam Antibiotics, Performance Liquid Chromatography with Ultraviolet Detection. J. Food Drug Analys..

[32] Nemutlu E., Kır S., Katlan D., Beksac M.S. (2009). Simultaneous multiresponse optimization of an HPLC method to separate seven cephalosporins in plasma and amniotic fluid: Application to validation and quantification of cefepime, cefixime and cefoperazone. Talanta.

[33] Pawliszyn J. (2000). Theory of Solid-Phase Microextraction. J. Chromatogr. Sci..

[34] Bojko B., Mirnaghi F., Pawliszyn J. (2011). Solid-phase microextraction: A multi-purpose microtechnique. Bioanalysis.

[35] Pfizer Ltd. (2013). Tazocin 4 g/0.5 g Powder for Solution for Infusion. Electron. Med. Compend..

[36] (2018). SPC Primaxin IV 500 mg/500 mg Powder for Solution for Infusion. https://www.medicines.org.uk/emc/product/1515.

